# Single harvesting in the all-inside graft-link technique: is the graft length crucial for success? A biomechanical study

**DOI:** 10.1007/s10195-016-0420-0

**Published:** 2016-07-19

**Authors:** Mattia Fabbri, Edoardo Monaco, Riccardo Maria Lanzetti, Dario Perugia, Matteo Guzzini, Luca Labianca, Andrea Ferretti

**Affiliations:** Orthopaedic Department and “Kirk Kilgour” Sports Injury Center, Sant’ Andrea Hospital, “La Sapienza” University of Rome, via di grottarossa, 1035-1039 Rome, Italy

**Keywords:** ACL, All-inside, Graft, Biomechanics

## Abstract

**Background:**

The all-inside graft-link technique for anterior cruciate ligament reconstruction is performed with two cortical suspension devices with adjustable loops on both femur and tibia. This technique requires meticulous graft preparation. The aim of this study was to biomechanically test three different graft configurations resulting from differences in initial graft length.

**Materials and methods:**

Thirty bovine digital extensor tendons were arranged in three different ways: “half-quadrupled”, “tripled” and “quadrupled”. The final graft length was 65–75 mm. The specimens were fixed vertical to the loading axis of a tensile testing machine. After a static pre-conditioning of 50 N for 5 min, a load to failure test was performed and data regarding the ultimate failure load (UFL), the stiffness and mode of failure were recorded.

**Results:**

The evaluation of UFL showed a significant differences between group means as determined by one-way analysis of variance (*F* = 21.92, *p* = 0.002). Post hoc comparisons showed a significantly better UFL of “tripled” (*p* = 0.007) and “quadrupled” preparations (*p* = 0.014) compared to the “half-quadrupled” configuration, with no significant differences between “tripled” and “quadrupled” grafts (*p* = 0.061). No significant differences were found when evaluating the stiffness between the groups. Failure occurred by tendon slippage across the suture in all specimens.

**Conclusion:**

The “quadrupled” tendon achieved the best UFL, with even the “tripled” configuration having sufficient biomechanical characteristics to withstand the loads experienced during early rehabilitation. For this reason, with a total semitendinosus length of less than 260 mm it could be better to “triple” instead of “half-quadruple” it to achieve better performance of the graft.

## Introduction

Anterior cruciate ligament (ACL) reconstruction has become one of the most common procedures performed by orthopedic surgeons. The success of a repair depends on several factors such as surgical technique, graft selection and biomechanical properties of the device used to fix the graft before integration [1, 2]. In recent years hamstring tendon grafts have become popular because of low donor-site morbidity and adequate biomechanical properties [3, 4]. Otherwise, there is still no agreement on the optimal fixation techniques, so several studies have focused on biomechanical properties of the most common femoral and tibial fixation implants [5–11]. Recently, a new anatomical, single-bundle, all-inside ACL reconstruction technique using a second-generation cortical suspension device with adjustable graft loop length on both femur and tibia was described. In this gracilis-sparing technique, the harvested semitendinosus is looped into four strands and linked, like a chain, to ACL femoral and tibial TightRope Reverse Tension devices (Arthrex^®^, Naples, FL, USA). With this device, the tension of the graft can theoretically be increased even after graft fixation, using the adjustable length of the graft loop. As well described by Lubowitz [13, 14], a meticulous preparation of the graft is critical for a successfully ACL all-inside repair. However, the graft length is strongly correlated with the total semitendinosus tendon length, which is not always sufficient to achieve a quadrupled graft. For this reason, other graft configuration techniques have been proposed [13, 14]. The aim of this study was to test and compare three different graft preparations resulting from differences in initial graft length, the hypothesis being that there would be no difference in the biomechanical performance of the three grafts.

## Materials and methods

Bovine digital extensor tendons were harvested from 15 hind limbs of 20-month-old bovines and tendons were stored at −22 °C and then thawed before use. They were kept moist until testing by wrapping in tissue paper soaked with Ringer’s solution and stored in sealed polyethylene bags.

The bifurcate tendon was divided into two halves and each single tendon was arranged in three different ways in order to reach a graft length between 65 and 75 mm, such as is needed for the surgical all-inside graft-link technique [13, 14].

**Fig. 1:**
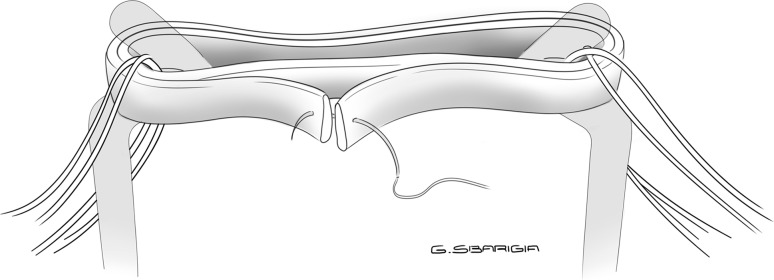
Quadrupled configuration: the tendon is quadrupled so that the free ends of the graft are passed on the same side of the loop and then whipstitched together

**Fig. 2:**
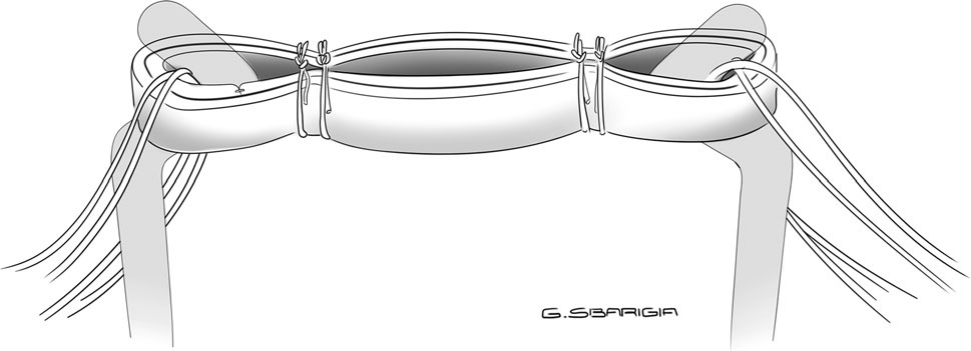
Quadrupled configuration: each wire is passed through each of the four strands of the graft on both sides, obtaining a “4 + 4” preparation

The first preparation consisted of a “quadrupled” graft: the tendon was looped and quadrupled so that the free ends of the graft were passed on the same side of the loop and then whipstitched together with a N.02 Fiberwire (Arthrex^®^) (Fig. 1). Next, two sutures were placed on the tibial side of the graft and two on the femoral side. Each stitch was passed through each strand of the graft, and the suture limbs were wrapped once around the bundles, creating a self-reinforcing suture noose when tied, in a buried-knot technique [13]. With this preparation, each wire was passed through each of the four strands of the graft on both sides, obtaining a “4 + 4” configuration (Fig. 2).

**Fig. 3:**
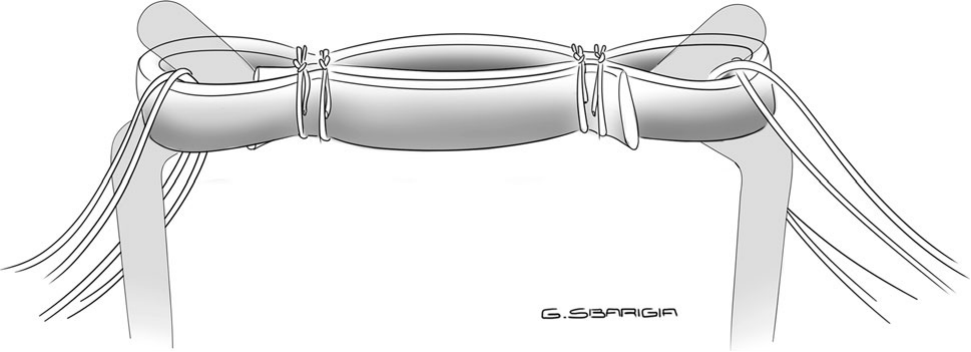
Tripled configuration: each wire is passed through each of the three strands of the graft on both sides, obtaining a “3 + 3” preparation

The second preparation consisted of a “tripled” graft: the tendon was looped and tripled so that the free ends were passed on different sides, then two sutures with N.02 Fiberwire were placed on both the tibial and the femoral sides of the graft, and secured with a buried-knot technique [13]. With this preparation, each wire was passed through each of the three strands of the graft on both sides, obtaining a “3 + 3” configuration (Fig. 3).

**Fig. 4:**
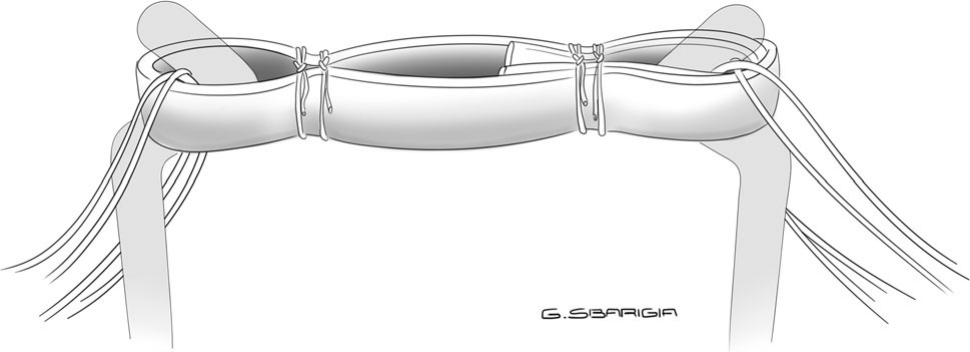
Half-quadrupled configuration: quadrupled loop on one side and doubled loop on the other, obtaining a “4 + 2” preparation

Finally, the third preparation consisted of a “half-quadrupled” graft: the tendon was wrapped around the hook of the graft-preparation station and the tendon’s free ends were held by hemostats so that they were passed on the same side of the loop. Two sutures with N.02 Fiberwire were then passed on each side and secured with a buried-knot technique [13], obtaining a quadrupled loop on one side and a doubled loop on the other one (“4 + 2” configuration) (Fig. 4).

Each construct was mounted and fixed on a tensile testing machine (model Z010, Zwick-Roell, Ulm, Germany) using two cylindrical metal rods directly connected to the load cell. The graft was vertical to the loading axis of the machine so that the force was applied in line with the graft, testing the worst load scenario. In order to stabilize the mechanical properties of the graft, a static pre-conditioning of 50 N was applied for 5 min, then a load to failure test was performed. Data regarding the ultimate failure load (UFL) and the stiffness of each specimen were recorded with Textexpert 8.1 software (Zwick-Roell) and evaluated with a load-displacement curve. The mode of failure of each construct was also recorded.

The effect size was calculated by eta-squared (*η* ^2^) = sum of squares (SS) between groups/total SS.

All the data were analyzed by a single blinded researcher. Computed *p* values were two-sided, and *p* < 0.05 was used to determine statistical significance. For all variables, normality of data was ascertained by the Kolmogorov–Smirnov test. Homogeneity of variance was estimated using Levene’s test. One-way analysis of variance (ANOVA) was performed on differences between group means. Post hoc analyses were performed using standard Tukey procedures with *α* correction for multiple comparisons. Statistical Package for the Social Sciences (SPSS) version 18 was used for calculations.

## Results

Ten specimens were tested for each graft preparation, resulting in a total of 30 tests performed.

All data were normally distributed (Kolmogorov–Smirnov test, *p* > 0.05) and between-group variances were equal (Levene’s test, *p* > 0.05). Tables 1 and 2 report the sample baseline characteristics. The evaluation of UFL showed statistically significant differences between group means with a large effect size as determined by one-way ANOVA (*F* = 21.92, *p* = 0.002, *η* ^2^ = 0.88). Post hoc comparisons showed significant differences in UFL between “half-quadrupled” and “tripled” preparations (*p* = 0.007) and between “half-quadrupled” and “quadrupled” graft preparations. There were no statistically significant differences between “tripled” and “quadrupled” graft preparations (*p* = 0.061) (Table 3).

**Table 1:** Baseline characteristics of the samples (UFL in N)

	Half-quadrupled	Tripled	Quadrupled
Mean	513.35	650.70	767.02
Standard deviation	55.27	27.41	53.19

**Table 2:** Baseline characteristics of the sample (stiffness in N/mm)

	Half-quadrupled	Tripled	Quadrupled
Mean	110.8	110.03	112.5
Standard deviation	14.72	8.2	9.6

**Table 3:** Comparison of UFL between groups and relative *p* values

	Mean difference	Standard deviation	95 % confidence interval of the difference		*p*
			Lower	Upper	
Half-quadrupled vs tripled	−137.35	40.88	−202.39	−72.30	0.007
Half-quadrupled vs quadrupled	−253.67	98.16	−409.87	−97.48	0.014
Tripled vs quadrupled	−116.33	79.64	−243.04	10.39	0.061

When evaluating the stiffness of the three groups, the statistical analysis showed no significant difference between the different graft preparations.

**Fig. 5:**
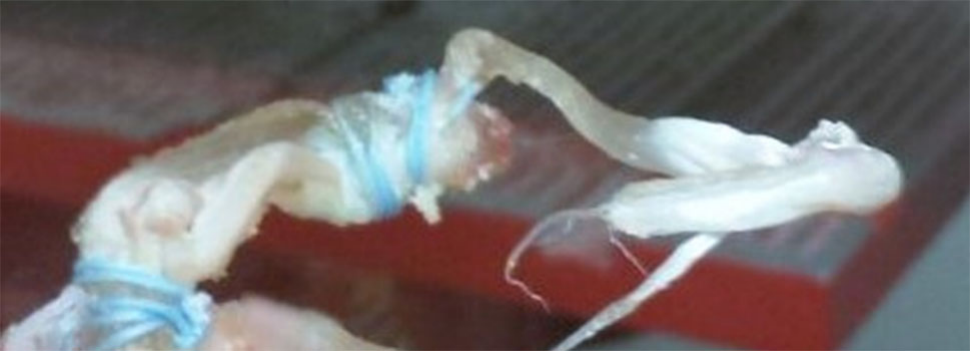
Mode of failure: tendon slippage across the suture (“quadrupled” configuration)

**Fig. 6:**
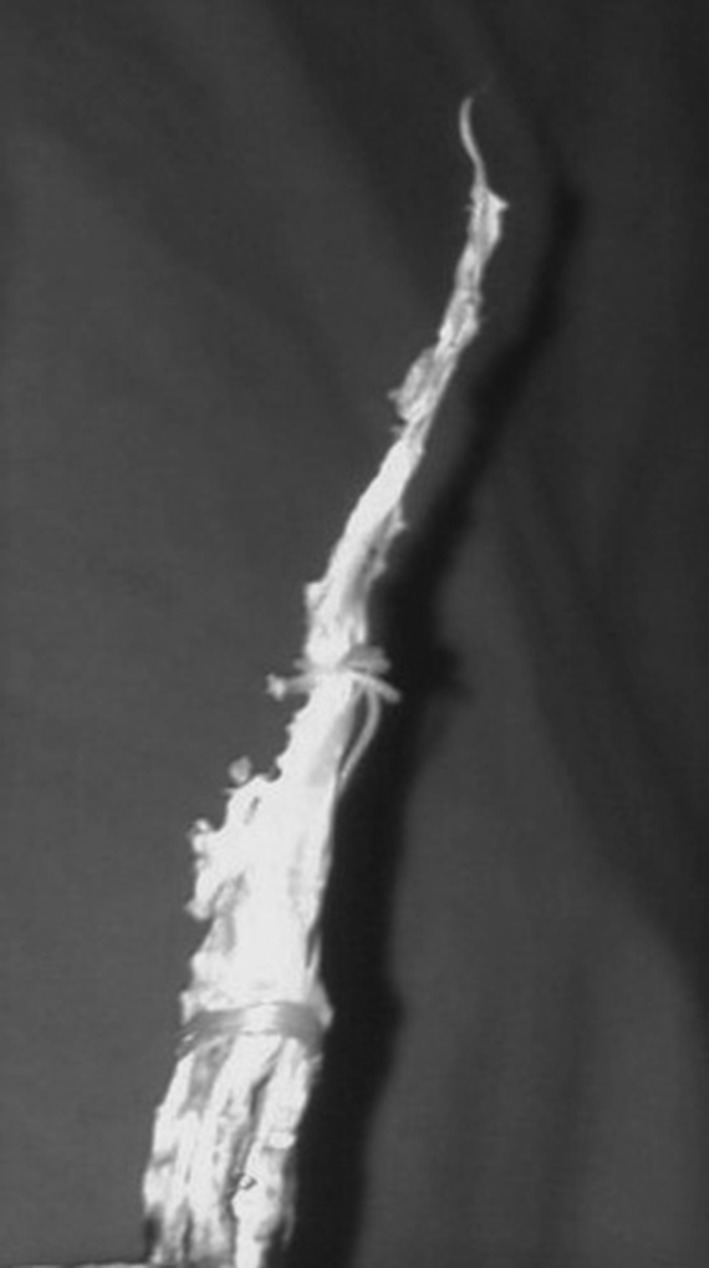
Mode of failure: tendon slippage across the suture (“tripled” configuration)

Failure occurred by tendon slippage across the suture in all specimens (Figs. 5, 6).

## Discussion

The most important finding of this study is that all the three graft preparations tested demonstrated sufficient UFL to withstand the repetitive loading forces that occur in the early postoperative rehabilitation period and during routine activities of daily living. It is estimated that those in vivo forces range from 67 to 454 N depending on the activities involved [12].

However, when evaluating UFL group means, statistical analysis showed a significant difference between the three graft preparations with a large size effect, with “tripled” and “quadrupled” grafts achieving better biomechanical performances. Thus, the hypothesis of the study was discarded.

As failure always occurred by slippage across the suture, we could speculate that, when the tendon is “tripled” or “quadrupled”, a better configuration is achieved to withstand loads because the suture is passed through more strands of the graft when compared to the “half-quadrupled” technique.

When considering the stiffness, we did not find differences between the three groups and this can be explained because the stiffness should be correlated more with the viscoelastic properties of the tendon itself rather than with the construct preparation.

The recently described all-inside graft-link technique using two TightRope Reverse Tension devices is an a ACL procedure which present some advantages such as lower morbidity of donor site, less postoperative pain and tensioning from both sides of the graft at any degree of extension [14, 15]. This technique is based on drilling two bone sockets on both the femoral and tibial sides using the Flipcutter drill (Arthrex^®^). Moreover, this technique also allows the surgeon to perform an anatomical ACL reconstruction in cases with a shorter depth of the femoral condyle because the graft completely fills the socket, which should be at least 20–25 mm to ensure bone graft incorporation. For all these reasons, graft length is crucial for ACL all-inside reconstruction to achieve complete filling of the graft on both tibial and femoral sockets with an adequate intra-articular portion. As well described by Lubowitz, meticulous graft preparation is crucial for a successful technique, so the author recommends the following graft characteristic: no greater than 270 mm in length, so that the final length when quadrupled is no more than 75 mm [13]. However, if the harvested graft has inadequate length, it is suggested that the graft be tripled or even to harvest the gracilis. Results from this study seem to demonstrate that, although the “quadrupled” tendon achieved the best UFL, the “tripled” configuration also had sufficient biomechanical characteristics to safely withstand loads experienced during early rehabilitation, while the “half-quadrupled” configuration demonstrated a lower strength. For this reason, with a total semitendinosus length of less than 260 mm it is better to “triple” instead of “half-quadruple” it, to achieve better graft performance.

Since this was an in vitro study, it has some limitations. First, we used animal tissues instead of human cadaveric specimens: bovine tendons were used because the stiffness and viscoelastic behavior are not significantly different from a human double-looped semitendinosus and gracilis graft [16] and they typically have cross-sectional diameters of 8 mm (long direction) and 5 mm (short direction), in agreement with the mean cross-sectional area of 43 mm^2^ reported by Noyes et al. [12] for a four-strand semitendinosus-plus-gracilis tendon graft. Second, as the specimens were frozen and then thawed before the test, this procedure could have altered their biomechanical properties, possibly affecting mode of failure and both UFL and stiffness [17]. Another limitation is that we did not test cyclic displacement because this is correlated more with the viscoelastic properties of the tendon than with the construct itself. Finally, as we tested only the suture technique as originally described by Lubowitz [13], the role of different suture techniques or graft augmentation still have to be determined in future studies. Moreover, further studies are needed to evaluate the biomechanical properties of the complete femur–graft–tibia complex with fixation devices using human cadaveric specimens, which is a configuration more similar to an in vivo ACL reconstruction technique. As a matter of fact, while biomechanical testing increases the validity of these results [7, 18, 19], it does not provide insight into the biological behavior of graft-tunnel healing after surgery that will ultimately determine the success or failure of the ACL reconstruction [20–22].

In conclusion, the results of this work have shown that all three graft configurations tested have sufficient UFL under the in vivo forces experienced during the early postoperative period, with better performance achieved when the graft is arranged in a “quadrupled” or even a “tripled” manner.
